# Increase in occurrence of large glacier-related landslides in the high mountains of Asia

**DOI:** 10.1038/s41598-021-81212-9

**Published:** 2021-01-15

**Authors:** Jia Liu, Yuming Wu, Xing Gao

**Affiliations:** 1 State Key Laboratory of Resources and Environmental Information System, Institute of Geographic Sciences and Natural Resources Research, Chinese Academy of Sciences, 11A Datun Road, Chaoyang District, Beijing, 100101 China; 2 University of Chinese Academy of Sciences, Beijing, 100049 China

**Keywords:** Climate sciences, Environmental sciences, Natural hazards

## Abstract

Globally, a large number of glaciers are retreating due to global warming. Along with climate change, glacial melting has been identified as one of the main triggers of landslide activity in high mountain areas. Evaluations of the triggered mechanism alone do not provide comprehensive insight into the overall impact of glacier accumulation and ablation on landslide-induced denudation. To investigate recent trends, we built landslide and glacier datasets for the HMA using a Landsat time-series covering the past 21 years (1998–2018). Landslides that may have been caused by major earthquakes were identified and removed, leaving an inventory that is used to explore changes that may be related to climate change. Our results show a shift in the frequency–area distribution that indicates an increasing trend of large landslides in the HMA over the last decade. A decline in glacier area is associated with the increase in landslide area.

## Introduction

Landslides, which can be triggered by events such as rainfall, earthquakes and freeze–thaw cycles, occur frequently in mountainous areas^1–4^. Due to climate change, hazards, such as glacier retreat, permafrost degradation and lake shrinkage, occur frequently in high altitude regions^5–7^, often leading to slope instability, and an increase in the occurrence of deep-seated landslides^8^.

Climate change has an indirect effect on landslides occurring at high altitudes by degrading permafrost^9^ and melting glaciers^10,11^ which may increase the magnitude and frequency of landslides^12^. Sediments, which tend to be accumulate on the receding edge of a glacier, may become unstable as the glacier recedes^13^. Glacial melting can also change precipitation patterns^14^, especially extreme rainfall, which tends to exacerbate slopes instability. Although observations of recent large landslides in some mountain regions lend support to these hypotheses regarding climate change and slope stability, the evidence is typically ambiguous. This is partly due to the lack of systematic records of historical landslides that can be compared with new landslide data, which is a problem in alpine and glacial environments in many parts of the world^15–17^.

This paper therefore investigates the spatial and temporal patterns of landslides in the high mountains of Asia (HMA) over a 20-year period (1999–2018) using a custom-derived glacier-related landslide dataset. Our aim is to investigate whether the size and frequency of landslides in this region have changed as the region has warmed. The study area includes the eastern Pamir, western Himalayas, Hindu Kush, Karakoram, and western Kunlun mountains within region 33.8°–39.5°N and 70.5°–78.7°E, and covers an area of over 600,000 km^2^. An inventory of landslide maps and glacier extent for the past 20 years was generated using Landsat imagery. Landslides potentially of a seismic origin were excluded from the inventory based on proximity in time and space to recorded major earthquakes.

The HMA region is typical of many high altitude regions. Glaciers are widely distributed, and as an abundant water resource, make an important contribution to the supply of water to millions of people in the surrounding areas. The glaciated areas of the HMA are more sensitive to temperature than those in low-lying plains^18^. Climate change predictions for these aera include more frequent extreme weather events, recession of the glaciers, and changes in water levels in glacial lakes^19^. Geologic hazards occur frequently in the HMA^20,21^, and landslides have the potential to affect important transportation corridors connecting neighboring Asian countries that have to cross these mountains.

## Data and methods

### Landslide inventory

Our primary dataset is a glacier-related landslide inventory, which can be very useful for risk assessment^22^. Some scholars have conducted field surveys in the HMA and generated previous landslide inventories^23^. However, these databases are mostly historical data without detailed and reliable timestamps. Remote sensing offers the possibility of addressing this data gap, by building detailed time-series of landslide inventories.

Landslides can be detected on satellite series by changes in land cover (vegetation distribution, rock outcropping exposure or soil degradation)^15,24^. Remote sensing satellite data with high resolution an extensive archive of historical acquisitions, and a generally vertical field of view that provides an almost planimetric perspective, are particularly valuable for generating time-series data. Sentinel and Landsat are sensors that have both used by scholars to study landslides^25,26^. Although the spatial resolution of Landsat is slightly coarser than that of Sentinel, Landsat provides a longer observation time. To avoid biasing our time series, with more landslides being identified from the more recent high-resolution imagery, we use only Landsat imagery.

For our study, Landsat imagery was obtained from the U.S. Geological Survey (USGS) EarthExplorer website. Images were obtained for each year for the period from 1998 to 2018. The Landsat data include images from the Thematic Mapper (TM), Enhanced Thematic Mapper (ETM+) and Operational Line Imager. The spatial resolution of these data is 30 m. The entire study area is covered by 17 Landsat path/row scenes. For each scene, cloud-free images at the end of summer (September through October) were selected. Additional images were selected during the winter season of each year so that each year's imagery includes one imagery at beginning or end of the year.

**Table 1:** Earthquakes that potentially triggered landslides in the study area.

Date	Latitude (°)	Longitude (°)	Epicenter	Magnitude
2015-12-07	38.21	72.77	104 km W of Murghob, Tajikistan	7.2
2015-07-03	37.45	78.15	97 km SE of Yilkiqi, China	6.4
2005-10-08	34.53	73.58	Pakistan	7.6
2002-11-20	35.41	74.51	Northwestern Kashmir	6.3

Landslides were manually identified by photo-interpretation of the scenes, and by comparing imagery between dates. Landslide scars and deposits resulting from landslides can be identified on Landsat imagery from contrasts in landform and land cover with the surrounding areas, and confirmed by lack of these distinctive features in the summer image of the previous year. The multi-temporal database was used to constrain the date of the landslide, which was labeled with the year the landslide is first visible. The digitization of the landslide polygons was performed in ENVI software. SRTM3 digital elevation data is used to calculate the aspect and slope of a landslide.

In the process of mapping landslides, we tried to map all the affected area of each landslide. However, the failed areas of some landslides were not clearly visible because of shadows or a lack of contrast between ground disturbed by the landslide, and undisturbed snow/ice or bare rock. This leads to the possibility that the landslide area may include the failure area and the runout area, or it may include only the runout area or the accumulation area.

**Figure 1:**
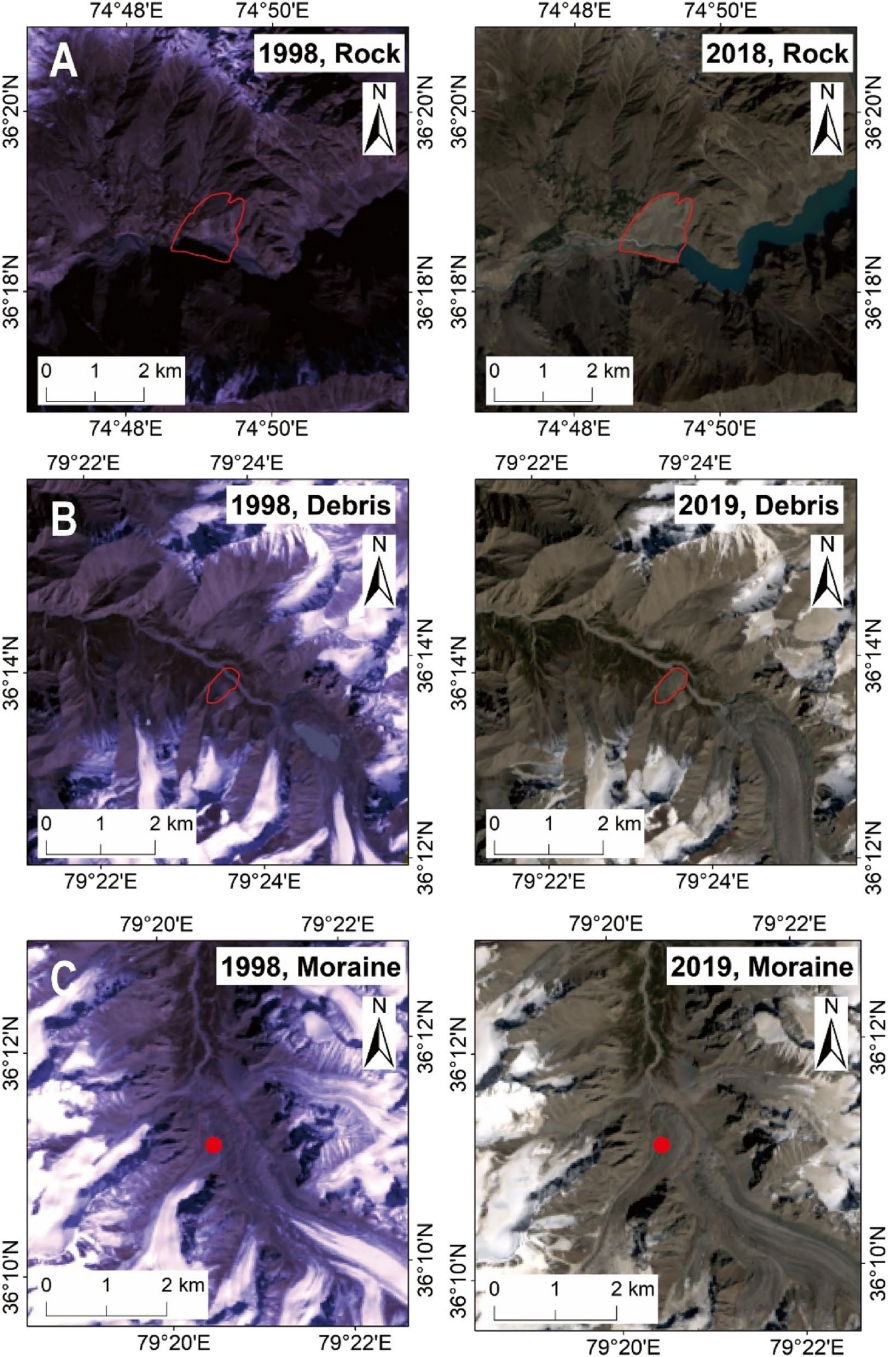
Comparison of three types of landslide source area. (**A**–**C**) are shows the landslides of rock, debris, and Moraine material types (Figures created using Arcgis10.2 https://www.esri.com/en-us/arcgis/products/arcgis-desktop).

We classified the types of landslides according to the criteria of Varnes (1998)^27^. Due to the limitation of Landsat image resolution, it is difficult to distinguish the type of movement. Therefore, we also used higher spatial resolution imagery available in Google Earth to supplement our Landsat interpretation. The landslide material types were rock, debris and moraine, as illustrated in Fig. 1.

**Figure 2:**
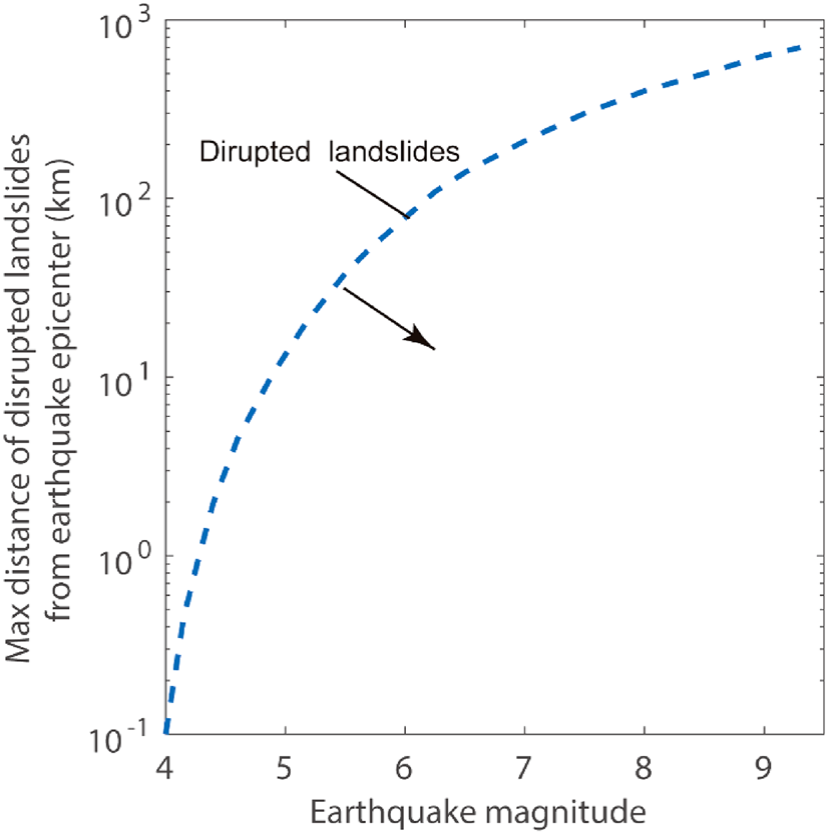
Variation in earthquake epicentral distance from disrupted landslides for earthquakes of varying magnitudes. The extent of black arrow points to is the distance range of disrupted landslides caused by the earthquake.

Because we focus on the possible effects of glacier retreat on landslides, we removed from our inventory landslides that could potentially have been triggered by earthquakes and that were more than 50 km from glaciers. Keefer^28^ has proposed a typical maximum distance for the occurrence of seismically-induced landslides from the epicenter; a distance that varies as a function of the magnitude of the earthquake and represented earthquakes (Fig. 2). We therefore used the USGS earthquake hazards website to identify all major earthquakes near or within the study area during the 20-year interval studied, and then removed from our inventory any landslide that could have occurred at the time of the earthquake and was located within the threshold of distance. We chose the disturbed landslide because it is the most sensitive to earthquakes, which means that the earthquake has a large impact distance on such landslides. We found four earthquakes (Table 1) and an associated four landslides that met the magnitude and distance criteria shown in Fig. 2. These landslides were removed from the inventory.

### Glacier mapping

The second major dataset that we generated for this study is a 20-year time series of maps of glacial extent for the study area. As with the landslide inventory, we used Landsat remotely sensed data. The Landsat sensors TM, ETM+ , OLI capture the distinctive spectral reflectance of snow and ice in the VNIR (visible and near-infrared) band and SWIR (shortwave infrared) band that is used to separate it from the surrounding terrain. Typical Landsat spectral measures include spectral band ratios such as TM3/TM5, TM4/TM5, and the NDSI (normalized difference snow index). In this study, we used the Landsat NDSI index to obtain glacial boundaries. The NDSI index is defined as:

1 $$NDSI = \frac{{\left( {{\text{Green}} - SWIR} \right)}}{{\left( {{\text{Green}} + SWIR} \right)}}$$

where Green and SWIR represent the values of the green and shortwave infrared bands for Landsat series images. We set 0.4 as the extraction threshold, which is commonly used by scholars to extract ice from remote sensing images^29^.

Spectral ratio methods are effective in delineating clean ice. However, seasonal snow, cloud and frozen lakes may be misclassified as glaciers and require manual editing of the classification. we therefore first classified ice using the NDSI. Then, we clipped our data based on the Randolph glacier inventory^30^, to reduce snowfall and cloud mass errors. Finally, manual interpretation was used to remove some errors associated with glacial lakes and around the edges of images.

### Probability-area distribution of landslides

A number of complex natural phenomena exhibit power-law frequency–area relationships, including earthquakes, which are considered a classic example of such phenomena. Landslides are thought to be another natural hazard that exhibits power-law frequency–area relationships under a wide variety of circumstances^31^. Some scholars question whether small landslides follow the power distribution, because small landslides sometimes evolve into larger landslides. There is also a problem that smaller landslides may be difficult to identify, or many individual small slides appear to be one large slide^32^. The main focus of this paper is medium to large landslides, so these concerns are not directly relevant to our work.

The probability-size distribution of landslides in the study area is given by

2 $$p\left( {A_{L} } \right) = \frac{1}{{N_{LT} }}\frac{{\delta N_{L} }}{{\delta A_{L} }}$$

where $p\left( {A_{L} } \right)$ is a probability density function, $\delta N_{L}$ is the number of landslides with areas between $A_{L}$ (km^2^) and $A_{L}$ + $\delta A_{L}$, $\delta A_{L}$ (km^2^) is based on a log scale and ${ }N_{LT}$ is the total number of landslides in an inventory. The frequency density of landslides, $f\left( {A_{L} } \right)$, is given by:

3 $$f\left( {A_{L} } \right) = N_{LT} p\left( {A_{L} } \right)$$

The frequency-size distribution of landslides in the study area was compared with those for various event magnitudes proposed by Malamud et al. (2004)^33^ to assess the nature of a landslide event. The $p$ value represents the probability density of landslides with area $A_{L}$ (km^2^) as follows:

4 $$p\left( {A_{L} :\rho ,a,s} \right) = \frac{1}{a\Gamma \left( \rho \right)}\left[ {\frac{a}{{A_{L} - s}}} \right]^{\rho + 1} exp\left[ { - \frac{a}{{A_{L} - s}}} \right]$$

where $\rho$ is a parameter controlling the power-law decay for medium and large landslides, $a$ (km^2^) is a parameter controlling the location of the maximum probability distribution, $s$ (km^2^) is a parameter controlling the exponential rollover for small landslides, and $\Gamma \left( \rho \right)$ is the gamma function of $\rho$. Malamud et al.^33^ also proposed a magnitude scale for a landslide event, $m_{L}$, as follows:

5 $$m_{L} = log_{10} \left( {N_{LT} } \right)$$

The combination of Eqs. (2)–(5) provides the frequency density of landslides linked to the magnitude scale of a landslide event.

For our study, the power-rate distribution was obtained using the R language.

## Results and discussion

### Factors influencing identification of landslides

Landslides can be identified in alpine and glacial regions by (1) high contrast compared to surrounding snow and ice, (2) the influence of landslide on river, and (3) lobate forms typical of rock-avalanche deposits^15,34^. Local variations in tone, texture or pattern, and the presence of lineaments can also be used to infer slope instabilities^35^. However, there are some factors that affect us to see the certain landslide in the identification process. The factors affecting landslide identification include landslide characteristics, snow cover, and the quality of remote sensing image.

Landslides are a complex movement process. Landslides include failure area, transportation area and accumulation area. Due to the influence of image quality (resolution, clouds, etc.) and the geological characteristics of the alpine region (glacier development, lack of vegetation), it is difficult to distinguish some slides which didn't cause optical characteristic variation in the image. They include some small area debris flow and moraine failure, and rock avalanches that have not formed significant accumulation. In our identification process, we found that the influence of landslide on river is beneficial to our identification work.

**Figure 3:**
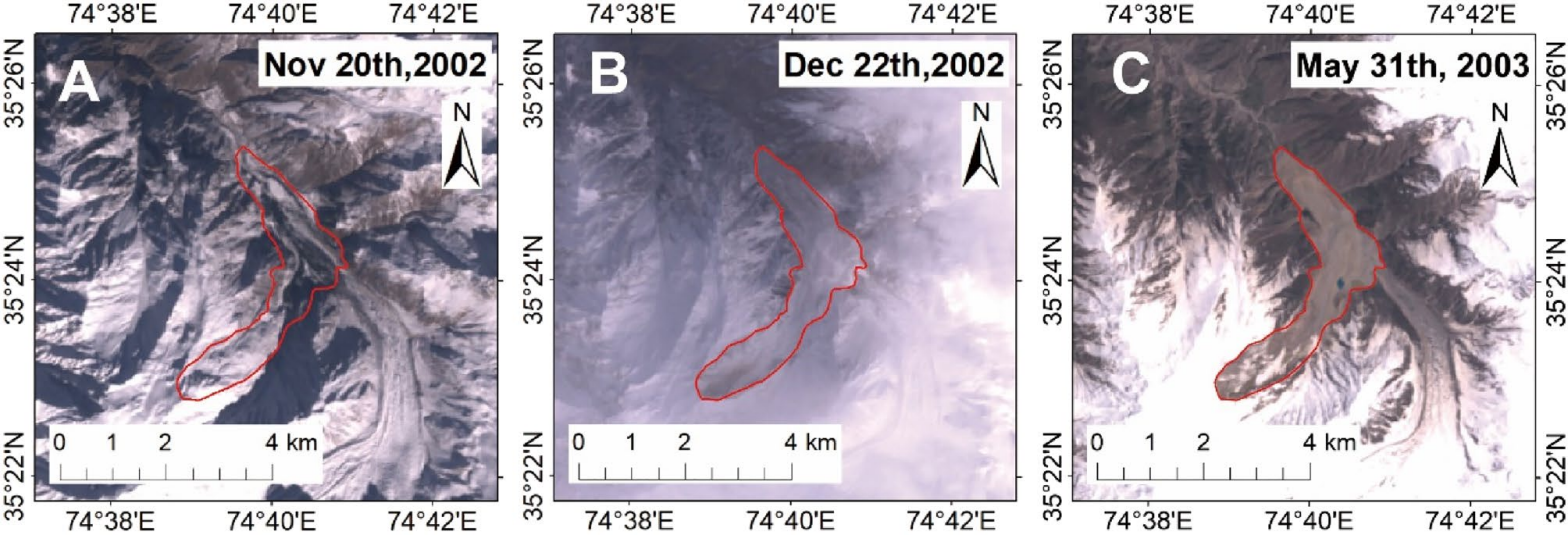
Comparison of landslides before and after being covered by snow. (**A**) is the scene before the landslide failed, (**B**) is the scene after the landslide failed with snow cover, (C) is the scene after the landslide failed without snow cover (Figures created using Arcgis10.2 https://www.esri.com/en-us/arcgis/products/arcgis-desktop).

The influence of external factors mainly comes from the influence of snow cover. According to our identification process, although a landslide occurs in winter and is covered with snow (Fig. 3A,B), it can be seen after the snow melts as the area is large or the accumulation is large (Fig. 3C). Coe et al.^15^ mentioned that the accumulation of landslides greater than 0.5 km^2^ would be obvious.

The quality problems of Landsat series satellites mainly come from cloud cover, seasonal snow cover and so on. We try to ensure a late summer and cloudless image every year to identify landslides. But sometimes it can't meet two conditions. Hence, we selected multiple images from the same year to identify the landslide. In particular, there is a problem with the black stripe error in the Lansat7 after it broke down in 2013. We think it is inevitable that we will miss some of the landslides that occur within these bands or most of them. At the same time, we can be sure that some large areas of the landslide, even if the impact of the strip, we can identify and determine its occurrence.

### Activity of landslides and climate change in the HMA

**Figure 4:**
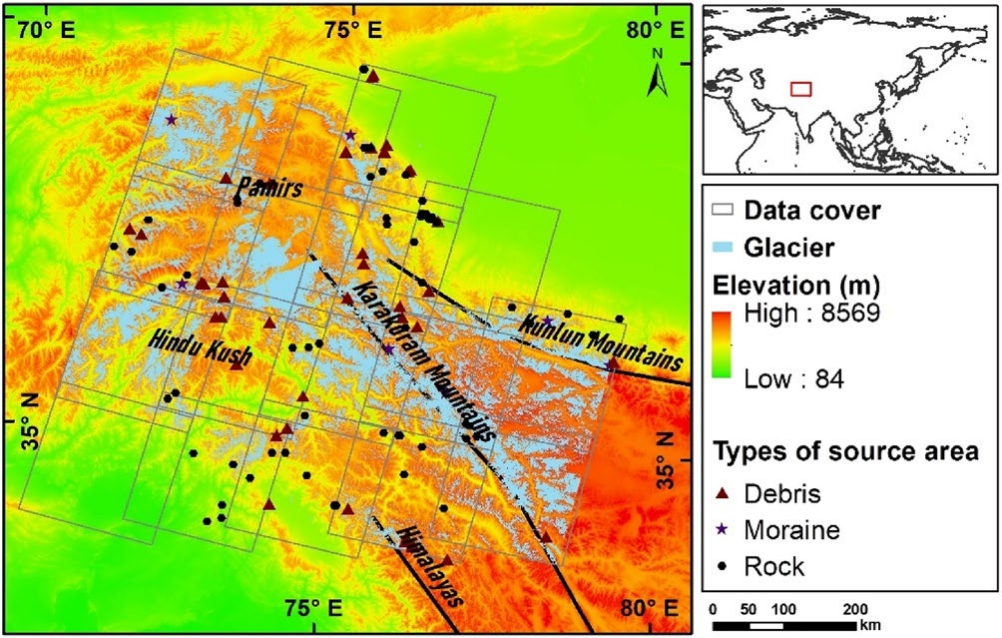
Study area. The gray rectangles represent the coverage of the Landsat images. Triangles, pentagons, and circles represent three types of landslide source area. The blue filled areas are glacier-covered areas (Figures created using Arcgis10.2 https://www.esri.com/en-us/arcgis/products/arcgis-desktop).

**Figure 5:**
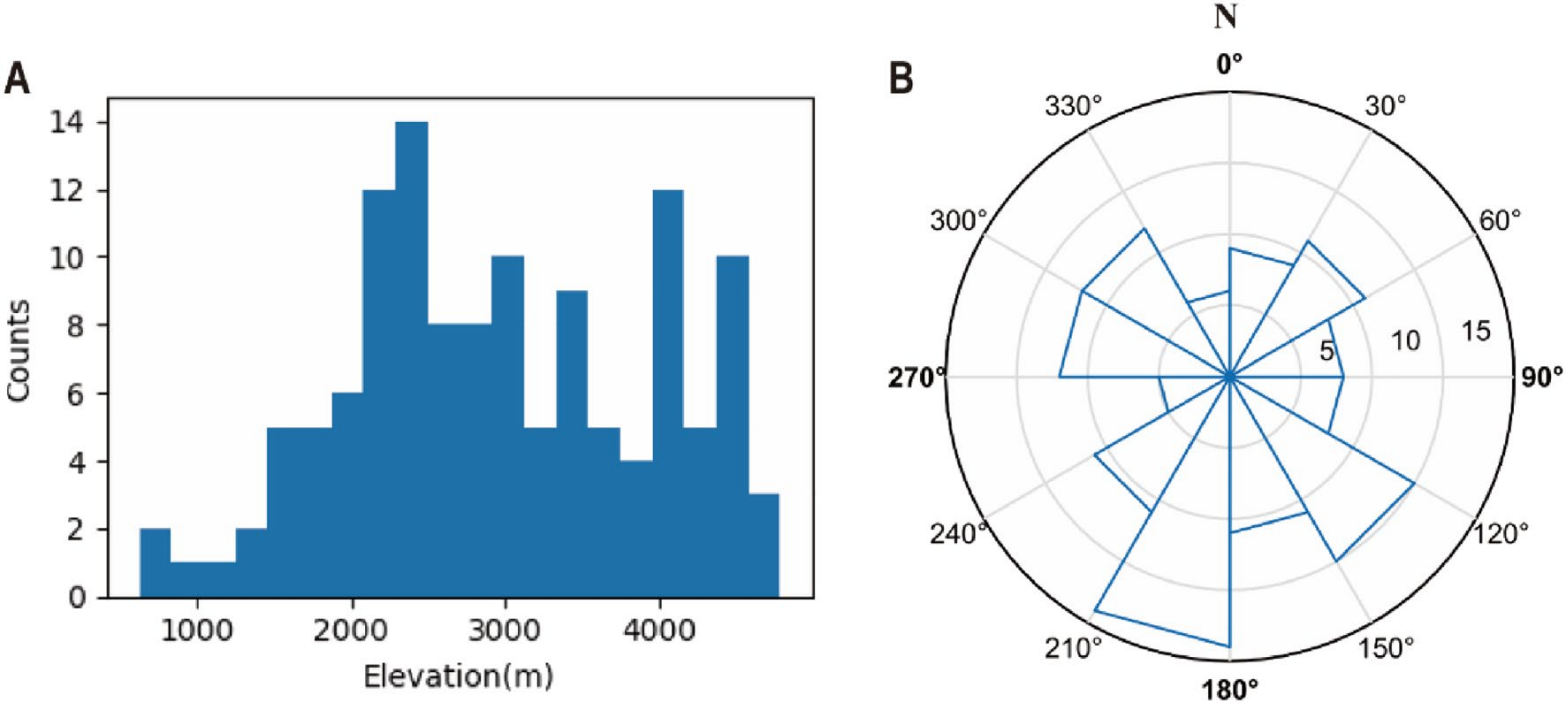
Variation in (**A**) elevations and (**B**) aspects for landslide from a 30-m DEM. Aspects are shown on a 360° Rose diagram. Numbers on circles are counts within that range.

A total of 127 landslides were detected in the Landsat images of the study area, covering the period from 1999 to 2018 (Fig. 4 and Supplementary Information). The landslides are mainly concentrated in the Karakoram Mountains, eastern part of the Pamir Mountains, western Himalayas and south of the Hindu Kush. Based on the source area the landslides were divided into three categories. A total of 72 rock landslides were identified, 45 debris landslides, and 10 moraine landslides. Table 2 and Fig. 5 summarize the landslide characteristics. The average area of the 127 landslides was approximately 58 ha, with 6.35 landslides occurring per year. The average elevation of the landslides is 2,966 m, and the average slope is 10.88° at the center point of the failure area. Most of the mapped landslides were at elevations more than 2,000 m above sea level (Fig. 5A). Landslide source areas had more slope directions (aspects) between 90° and 270° (i.e., southwest- to southeast-facing slopes, Fig. 5B).

**Table 2:** Landslide inventory summary characteristics.

Year	1999–2018
Total number of landslides	127
Total landslide area (m^2^)	73,811,719
Landslide mean area (m^2^)	581,194
Minimum landslide area (m^2^)	15,548
Maximum landslide area (m^2^)	7,801,274
Mean slope value(°)	10.88
Mean elevation(m)	2966

The landslides of rock type are widely distributed in the HMA, and concentrated in the western Kunlun Mountains, although they also occur frequently in the Hindu Kush and the western Himalayas. Debris landslides are mainly distributed in the West Kunlun Mountains and the Hindu Kush, with additional occurrences in the Pamirs and western Himalayas. As expected, morainic landslides occur at higher elevations, including the Karakorum Mountains, the western Himalayas, the Kunlun Mountains, and the Pamirs, at an average elevation of 4,139 m. We also analyzed the distribution of large landslides, defined as landslides that affected areas greater than 2 km^2^. A total of three large landslides were found in the Pamirs, four in the western Himalayas, and two in Karakorum.

**Figure 6:**
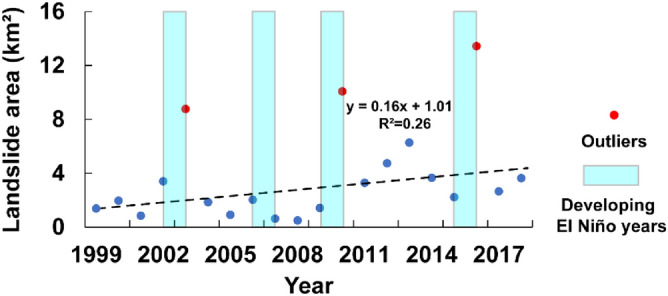
The orange scatterplot shows the temporal distribution of landslide area; blue shaded areas are developing El Niño years^38^. Red dots are outliers based on Thompson’s Tau^37^. The black dotted line is a linear-fit trendlines line of landslide area without outliers.

A graph of annual landslide area is show in Fig. 6. The Thompson Tau method^36^ is used to find the outliers with the critical probability alpha value is 0.01. The result indicates that the landslide area points in 2003, 2010 and 2016 are outliers, as shown in Fig. 6. After removing the outliers, linear-fit line (R^2^ = 0.26) shows that the annual landslide area in the HMA are increasing (Fig. 6).

The area of landslides was greatest in 2016. The average annual temperature was unusually high during this year, and the glacial area had notably declined in the previous 3 years (Fig. 8E). To provide additional insight into the impact of climate change on landslides, we compared the annual landslide area data with annual El Niño data for the study years^37^. We found that the landslide area outliers tend to coincide with El Niño years. The one exception is 2006, an El Niño year with no associated peak in landslide area. However, 2006 was associated with generally lower average temperatures in Fig. 8E. Overall, however, the time-series of landslide area, and particularly the outliers, appears to show periodicity, and the trend in landslide area is increasing. Scientists have hypothesized that as El Niño becomes more frequent and stronger, alpine glaciers are also being affected^38^. Warmer temperatures caused by El Niño can accelerate melting of glaciers, and the increase of rainfall also increases the conditions that favor the occurrence of landslides.

### Shifts in landslide frequency–area distribution

To investigate whether landslide characteristics have changed over the past 20 years, we divided the landslide data set into two datasets: landslides that occurred before 2009, and those that occurred in 2009 or later. Table 3 shows that both the number of landslides and the average area of landslides increased in the second 10 year period. The difference in the average slope of the landslides was not significant.

**Table 3:** Comparison of landslide inventory characteristics.

Year	1999–2008	2009–2018
Total number of landslides	42	85
Total landslide area (m^2^)	22,342,316	51,469,403
Landslide mean area (m^2^)	531,959	605,522
Minimum landslide area (m^2^)	43,931	15,548
Maximum landslide area (m^2^)	7,801,274	5,481,502
Mean slope value (°)	9.34	11.35
Mean elevation (m)	3115	2893

The power distribution of landslide data in the study area over the entire 20 years of the landslide inventory has a decay factor ρ of approximately 1.14, which is in the normal range of previous studies. Although there is some variability in the findings of previous research, most prior landslide data sets follow noncumulative power-law frequency statistics, and the range of ρ values is approximately 1.5 ± 0.5^32^. However, it is important to note that previous studies have obtained different power distributions, fitting different tail attenuation coefficients. Furthermore, the data sets used in these studies come from different environments and the associated earthquakes are assumed to have been triggered by different factors, including rainfall, earthquakes and snowmelt.

**Figure 7:**
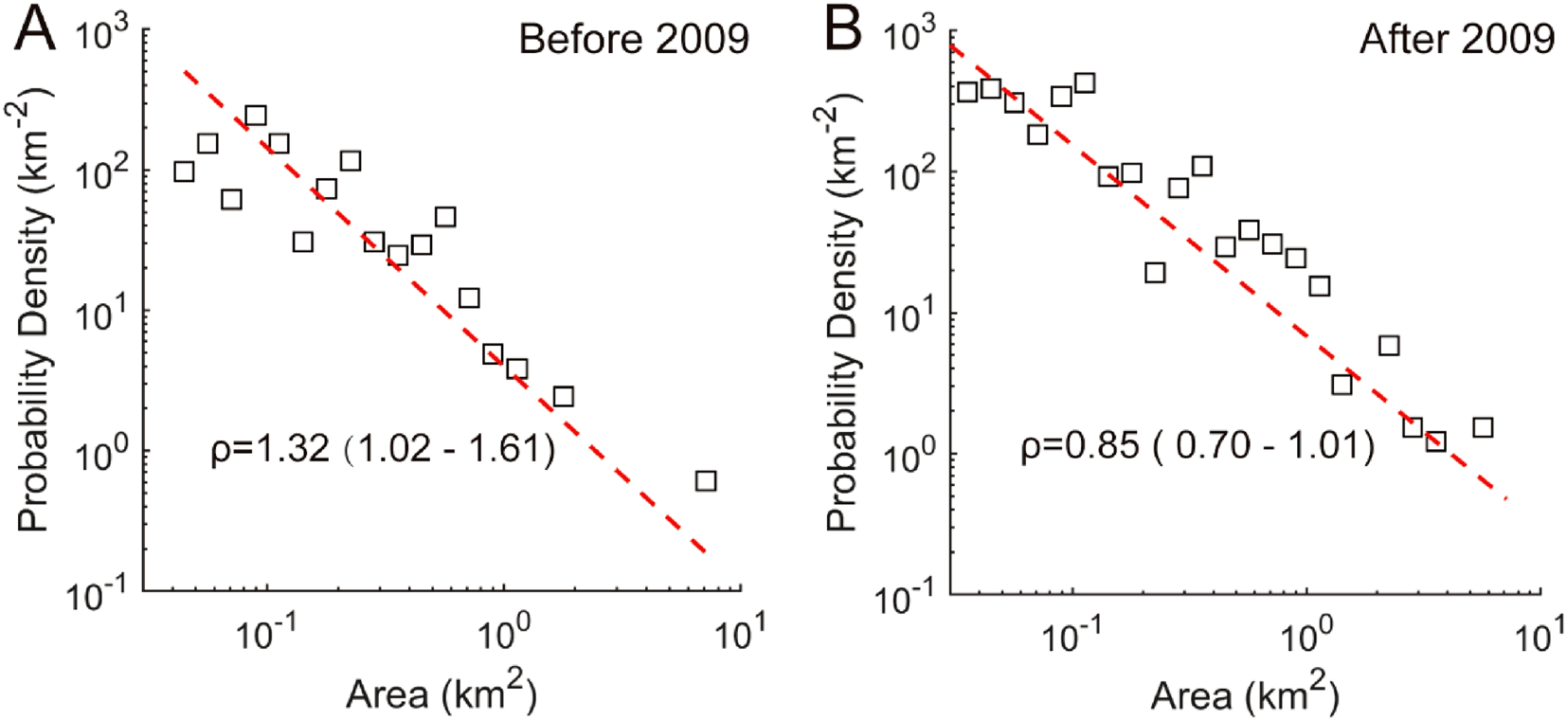
Comparison of frequency–area distribution for the 10 year period before 2009 and for 2009 and later. (**A**) is the frequency–area distribution for the 10 year period before 2009 with the decay factor ρ (1.32, 95%CI: 1.02–1.61). (**B**) is the frequency–area distribution for 2009 and later with the decay factor ρ (0.85, 95%CI: 0.70–1.01).

The power-rate distribution for the landslides separated into the two 10-year periods is shown in Fig. 7. Both periods show a distinct power distribution. The decay factor in the second 10 year period (ρ = 0.85, 95%CI: 0.70–1.01) is smaller than that of the first 10 year period (ρ = 1.32, 95%CI: 1.02–1.61). This indicates that the probability of large and medium-sized landslides in the second 10 year period is higher than the first period. The confidence intervals of the two values of ρ do not overlap, and thus the difference is statistically significant.

### Relationship between landslides and glaciation

For the entire study area, we used the Randolph glacier outlines as a mask to minimize misclassification due to factors such as seasonal snow. However, seasonal snow can also lead to errors in the calculations. In order to evaluate the error associated with the glacier delineation, we compared automatic extraction of glacial area with a semi-automatic approach that was regarded by scholars as a relatively accurate method^39^. The semi-automatic method uses the ratio threshold method to generate approximate results, when are then modified by hand, using visual interpretation by an expert interpreter. We selected data from 3 regions for data verification (Table 4). The calculated difference is less than 10%, and the average difference is about ± 6.3%. For our study, we think the error of the glacier area results is acceptable.

**Table 4:** Comparison of frequency–area distribution characteristics based on semi-automatic and automatic glacial mapping.

Region	(Upper left coordinates, Lower right coordinates)	Area from semi-automatic approach (km^2^)	Area from automatic approach (km^2^)	Difference between semi-automatic and automatic (%)	Acquisition year
Pamirs	([72.30, 39.56], [73.19, 38.19])	3636.2	3956.9	8	2009
Kunlun Moutians	([75.01, 38.40], [76.37, 36.48])	2927.3	2879.5	− 2	2014
Karakoram Moutians	([77.22, 35.43], [78.44, 33.80])	3674.5	4012.8	9	2000

The glacier area in the HMA has shown a downward trend over the past 20 years. Before 2005, the glacier area decreased and increased repeatedly, which may be related to the monsoon climate. After 2005, the area of glaciers decreased significantly. The trend of glacial degradation over the past decade was significantly stronger than that in the previous decade.

**Figure 8:**
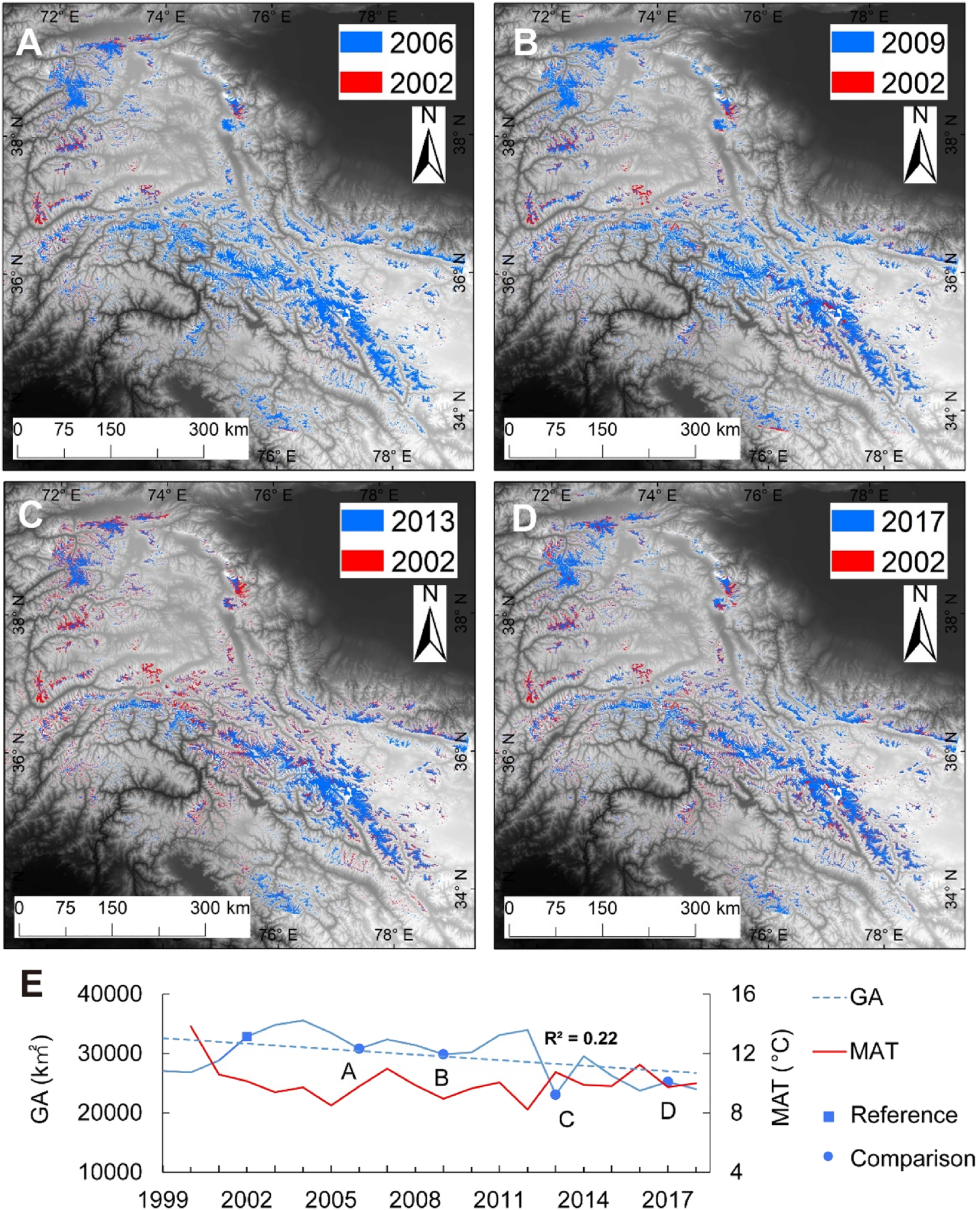
Comparison of glacial area in the study region for selected years. Panels (**A**–**D**) show glacial extent in 2002 and four other years (2006, 2009, 2013 and 2017, respectively). In each case, red indicates glacial extent lost during the time indicated, blue indicates glaciers present at both times. Panel E summarizes the overall trends in glacial area (GA) and mean annual temperature (MAT) during 20 years studied (Figures created using Arcgis10.2 https://www.esri.com/en-us/arcgis/products/arcgis-desktop).

For a comparison of the glacial extent between years, we chose the remote sensing image from 2002 as the basic reference because the imaging times for the mosaic image in 2002 were not very different. We selected the glacier area in four subsequent years (2006, 2009, 2013 and 2017) to compare with the reference to analyze the change in glaciers in the study area (Fig. 8A–D). We found that except for the Karakoram Mountains, which exhibited only small areas of glacial retreat over the past 2 decades, the retreat in the other areas was more extensive. An anomaly of the Karakoram Mountains was found due to the relatively high elevation^40^. In addition, climate anomalies and the strong summer monsoon climate in the region have brought additional moisture to the Karakoram Mountains, leading to increased snow^41^.

Figure 8E summarizes the total area of glacial extent of the 20-year period studied. The figure also shows the mean annual temperature, averaged over the region. MAAT was calculated based on MODIS surface temperature product MOD11A2, which has a spatial resolution of 1000 m and a temporal resolution of 8 days.

The HMA glacial area has had a downward trend over the past 20 years (Fig. 8E). Looking at the record in more detail, the glacial area decreased prior to 2005, and then increased, possibly in response to variations in the associated region’s monsoon climate. After 2012, the area of glaciers decreased notably. The glacial area showed little no overall change in the first decade, and a notable decline in the second decade. The temperature record does not show evidence of warming over the period 2000 to 2018. The year 2000 was unusually warm compared to the subsequent 18 years. Even excluding 2000, there is no clear evidence of a warming trend over the remaining years.

**Figure 9:**
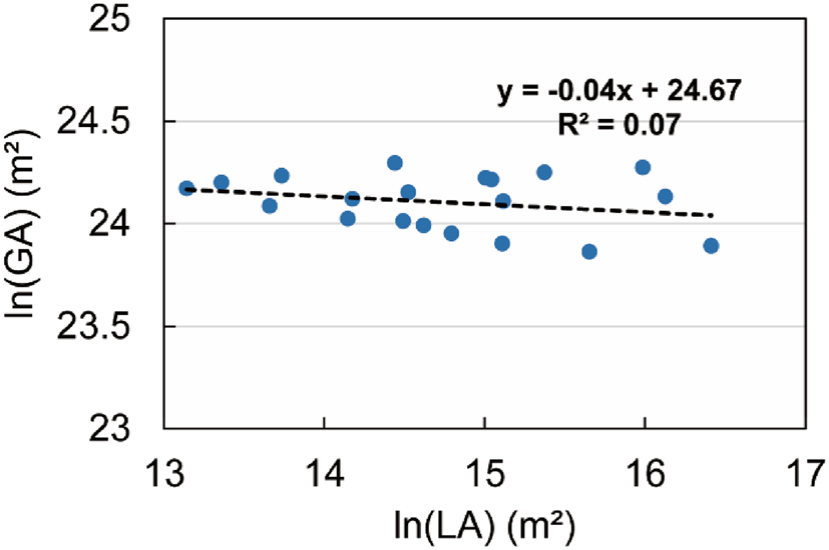
Graph of landslide area and glacier area. GA represents glacier area, and LA represents landslide area, and ln represents the natural log. The black dotted line is the linear trend line.

However, glacial area can be assumed to be an environmental proxy that integrates climate over time and space. Glacial area could therefore be a useful predictor of slope stability. Glacier area does indeed show a negative association with landslide area (Fig. 9), indicating landslide area is increasing as glacial area declines. Glacial retreat is an indicator of climate change, and our results support the hypothesis that warming of the region is associated with an increase in landslide occurrence. However, the small number of samples and the scatter indicate notable uncertainty, and the association therefore requires further research.

## Conclusion

In this work, we generated an inventory of 127 landslides in the HMA using Landsat data covering the 2 decades between 1999 and 2018. Annual glacial area maps for the same 20 years were also generated. In addition to the impact of image resolution, the rock surface and ice cover make landslide identification difficult in the HMA, which may lead to the number of landslides being underestimated. Nevertheless, the landslide and glacial area maps provide detailed spatial and temporal information. Based on our custom inventories, we studied the interaction between landslides and glaciers in the study area. The following major conclusions can be drawn from this work:

1. In the HMA, landslides are widespread. Over the past 2 decades, the area affected by landslide disasters has increased. Both the number of landslides and the affected area was larger during the most recent 10 years (2009–2018) compared to the previous 10 years (1999–2008). The area of landslides was anomalously high in three out of four El Niño years.
2. Landslides in the HMA follow power-law (fractal) frequency statistics. The attenuation coefficient of the 10 years from 2009 onwards decreased compared to the previous 10 years, indicating that the probability of occurrence of large landslides has increased.
3. Glacial area in the HMA has shown an overall downward trend over the past 20 years. Except for the Karakoram Mountains, most glaciers throughout the study area have been retreated. As the glacial area decreases, the area of annual landslides has increased.

In summary, retreat of glaciers in the HMA of 20 years appears to be associated with more frequent landslides, and larger landslides. A weak negative correlation between annual landslides and glacial areas is evident. Although local influences may have triggered landslides in the HMA, glacial retreat may be a useful proxy for aspects of climate that control slope stability. If climate warming continues, the area of glaciers will further decrease. As a result, the probability of large landslides in the HMA may continue to increase.

## Supplementary Information

Supplementary Information.
